# Lack of *ApoE* inhibits ADan amyloidosis in a mouse model of familial Danish dementia

**DOI:** 10.1016/j.jbc.2022.102751

**Published:** 2022-11-25

**Authors:** Anllely Fernandez, Maria-Teresa Gomez, Ruben Vidal

**Affiliations:** 1 Department of Pathology and Laboratory Medicine, Indiana University School of Medicine, Indianapolis, Indiana, USA; 2 Stark Neurosciences Research Institute, Indiana University School of Medicine, Indianapolis, USA

**Keywords:** ApoE, FDD, ADan, neurodegeneration, amyloid, Aβ, amyloid-β, AD, Alzheimer disease, CAA, cerebral amyloid angiopathy, CCP, classical complement pathway, FBD, familial British dementia, FDD, familial Danish dementia, Th-T, thioflavin-T, Th-S, thioflavin-S

## Abstract

The Apolipoprotein E-ε4 allele (APOE-ε4) is the strongest genetic risk factor for late onset Alzheimer disease (AD). ApoE plays a critical role in amyloid-β (Aβ) accumulation in AD, and genetic deletion of the murine *ApoE* gene in mouse models results in a decrease or inhibition of Aβ deposition. The association between the presence of ApoE and amyloid in amyloidoses suggests a more general role for ApoE in the fibrillogenesis process. However, whether decreasing levels of ApoE would attenuate amyloid pathology in different amyloidoses has not been directly addressed. Familial Danish dementia (FDD) is an autosomal dominant neurodegenerative disease characterized by the presence of widespread parenchymal and vascular Danish amyloid (ADan) deposition and neurofibrillary tangles. A transgenic mouse model for FDD (Tg-FDD) is characterized by parenchymal and vascular ADan deposition. To determine the effect of decreasing ApoE levels on ADan accumulation *in vivo*, we generated a mouse model by crossing Tg-FDD mice with *ApoE* KO mice (Tg-FDD^+/−^/ApoE^−/−^). Lack of ApoE results in inhibition of ADan deposition up to 18 months of age. Additionally, our results from a genetic screen of Tg-FDD^+/−^/ApoE^−/−^ mice emphasize the significant role for ApoE in neurodegeneration in FDD *via* glial-mediated mechanisms. Taken together, our findings suggest that the interaction between ApoE and ADan plays a key role in FDD pathogenesis, in addition to the known role for ApoE in amyloid plaque formation in AD.

Mutations in the *BRI*_*2*_ gene (also known as *ITM2B* (1)), located on the long arm of chromosome 13, cause the autosomal dominant neurodegenerative diseases familial British dementia (FBD), familial Danish dementia (FDD), and Chinese dementia (2, 3, 4). Point mutations in the stop codon of *BRI*_*2*_ cause FBD and familial Chinese dementia (2, 4), while FDD is caused by a 10-nucleotide duplication insertion (*BRI*_*2*_*795-796InsTTTAATTTGT*) in the 3′-end of the coding region of the *BRI*_*2*_ gene (3). FDD, originally named heredopathia ophthalmo-oto-encephalica because of the presence of cataracts, hearing problems, and neurological disease, was first described in members of a single Danish family in the Djursland peninsula (5, 6). Neuropathologically, FDD is characterized by the presence of cerebral amyloid angiopathy (CAA) in vessels of the retina and leptomeninges as well as in vessels of the gray and white matter of the central nervous system (3, 7). Amyloid plaques are found in the hippocampus, with abnormal neurites in the vicinity of blood vessels with amyloid. Neurofibrillary tangles composed of tau paired helical filaments and straight filaments (3, 7) are also found. Recent cryo-electron microscopy (cryo-EM) work showed that paired helical filaments and straight filaments in FDD and FBD are structurally identical to those seen in Alzheimer disease (AD) (8).

The BRI_2_ protein contains 266 amino acids and belongs to a family of integral type II single transmembrane domain proteins (1, 9). BRI_2_ is produced as a pro-protein with a pro-peptide sequence that is cleaved by pro-protein convertases (PCs) between the BRI_2_ ectodomain that releases the ADan amyloid in FDD (9) (Fig. 1). A transgenic animal model for FDD (Tg-FDD) expressing the Danish mutant form of human *BRI*_*2*_ under the control of the mouse prion protein promoter shows significant vascular and parenchymal ADan deposition, amyloid associated gliosis, intracellular and extracellular deposition of oligomeric forms of ADan, as well as tau immunoreactive deposits in the neuropil (10). A double transgenic mouse model generated by crossing Tg-FDD mice with mice expressing human 4-repeat P301S mutant tau shows significant enhancement of tau deposition and decrease in synaptophysin levels, suggesting that ADan and amyloid-β (Aβ) may share similar pathogenic pathway(s) (11).

**Figure 1:**
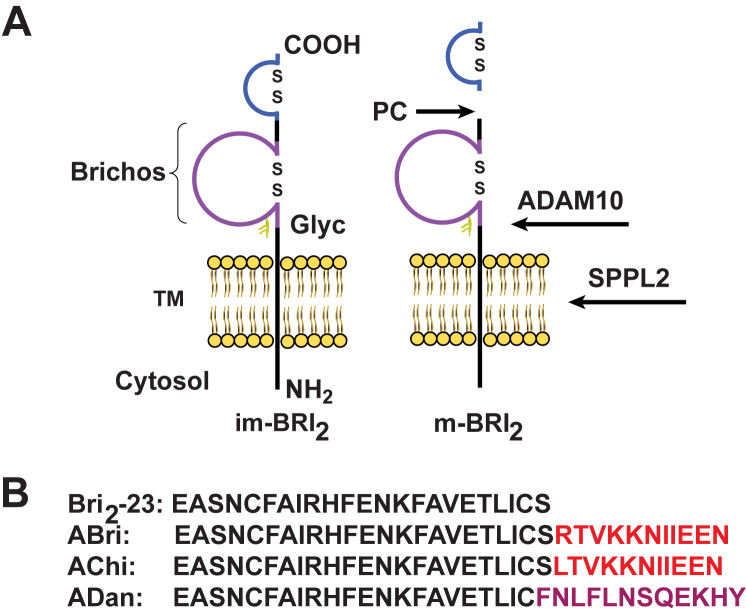
**Proteolytic processing the type-II single transmembrane (TM) domain BRI**_**2**_**protein.***A*, processing by ADAM10 in the ectodomain of BRI_2_ releases the BRICHOS domain (∼100 amino acid (aa)) and an N-terminal fragment, which the subject of additional proteolysis by SPPL2. Disulfide-bonded loops in the BRICHOS domain and in the carboxy terminus of BRI_2_ (aa 5 and 22 of the Bri_2_-23 peptide) are indicated as well as the single N-glycosylation site (Glyc) at position 170. *B*, cleavage of the pro-protein (or immature protein, im-BRI_2_) by pro-protein convertases (PCs) generates the 23 aa peptide (Bri_2_-23) and a mature form of BRI_2_ (m-BRI_2_). Processing of the FBD, FDD, and FCD forms of BRI_2_ by PCs releases the 34 aa amyloid peptides (ABri, ADan and AChi) in FBD, FDD, and FCD, respectively. FBD, familial British dementia; FCD, familial Chinese dementia; FDD, familial Danish dementia.

Genetic analyses have shown that the apolipoprotein E (*ApoE*) gene is the strongest genetic risk factor for developing late-onset AD (12, 13). Three alleles of *ApoE* (ε2, ε3, and ε4) exist, which generate three different ApoE proteins (ApoE2, ApoE3, and ApoE4) that differ by only one or two amino acids at positions 112 and 158, which seem to modify the structure and function of ApoE (14). The influence of ApoE on AD risk occurs in an isoform-dependent manner with the ε4 allele as the strongest genetic risk factor for AD (15, 16). Using antibodies against ApoE, it was observed that ApoE immunoreactivity was associated with amyloid deposits in AD and Creutzfeldt–Jakob disease (17), as well as in other cerebral and systemic amyloid diseases (18, 19), suggesting a more general role for ApoE in amyloid diseases as an amyloid catalyst or “pathological chaperone” (18). Genetic deletion of the murine *ApoE* gene (which is expressed as a single isoform) in mouse models overexpressing mutant forms of the human Aβ precursor protein (AβPP) results in inhibition of Aβ deposition or in a decrease in amyloid plaques (20, 21, 22, 23, 24, 25, 26, 27). The effect of ApoE on amyloid deposition seems to be dose dependent since a significantly lower number of amyloid plaques is seen in mice hemizygous for *ApoE* (20, 21, 24, 28, 29, 30), with astrocytic ApoE3 and ApoE4 having a major role on the deposition and accumulation of Aβ in amyloid plaques (31). A similar effect of ApoE in other cerebral or systemic amyloidoses has not been experimentally demonstrated.

Analysis of FDD knock-in mice (FDD_KI_ mice), which do not develop amyloid deposits, crossed with human *ApoE-ε3* and *ApoE-ε4* targeted replacement mice suggest that the FDD mutation may differentially affect learning and memory in *ApoE-ε4* carriers and noncarriers (32). Herein, we characterized the impact that ApoE has on amyloid pathology in a FDD transgenic mouse model, a model of cerebral ADan amyloidosis. We found that Tg-FDD mice on the murine *ApoE* KO background (Tg-FDD^+/−^/*ApoE*^*−/−*^) do not deposit ADan amyloid. We also observed accumulation of mature and immature forms of the BRI_2_ precursor protein in the hippocampus of Tg-FDD^+/−^/*ApoE*^*−/−*^ mice, which is not seen in sex/age matched Tg-FDD mice carrying the murine *ApoE* allele, and a significant change in the expression profile of genes related to autophagy, vesicle trafficking, angiogenesis, and activation of microglia. Our results suggest that apart from influencing amyloid plaque formation in AD, the interaction between ApoE and ADan plays a key role in FDD pathogenesis.

## Results

### ADan aggregation in the presence of different ApoE isoforms

**Figure 2:**
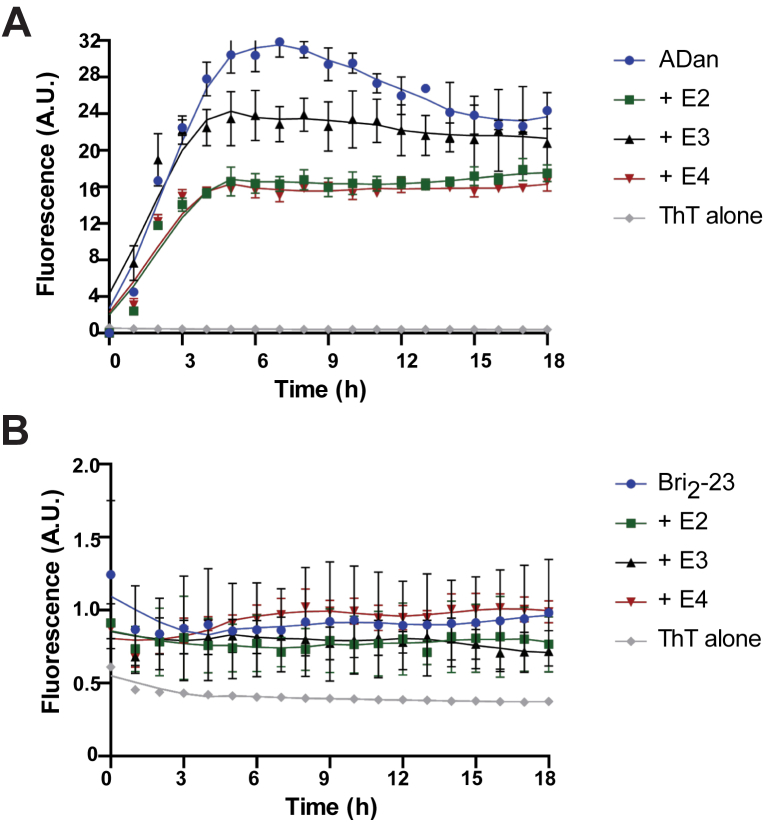
**Thioflavin-T (Th-T) binding assay.***A*, Th-T binding of ADan (30 μM) alone and in the presence of ApoE2, ApoE3, and ApoE4 (300 μM). *B*, Th-T binding of Bri_2_-23 (30 μM) alone and in the presence of ApoE2, ApoE3, and ApoE4 (300 μM). Fluorescence is expressed in arbitrary units (A.U.). All data are expressed as mean ± SD. Experiments were repeated three times.

The interaction between ADan and ApoE variants was assessed by monitoring ADan aggregation in the presence of 300 nM recombinant ApoE2, ApoE3, or ApoE4 using a thioflavin-T (Th-T)–binding assay. As a control, the WT Bri_2_-23 peptide was also analyzed. ADan aggregation kinetics at pH 7.2 displayed a sigmoidal curve shape. After 6 h, a peak in ADan aggregation was observed. The addition of the three different ApoE isoforms led to a pronounced decrease of Th-T incorporation, leading to lower plateau levels, without modifying lag-phases (Fig. 2*A*). No aggregation of the Bri_2_-23 peptide was observed under the same conditions over the entire time course of the experiment (Fig. 2*B*). Presence of the different ApoE isoforms had no effect on the aggregation of the Bri_2_-23 peptide.

### ApoE KO and ADan deposition *in vivo*

**Figure 3:**
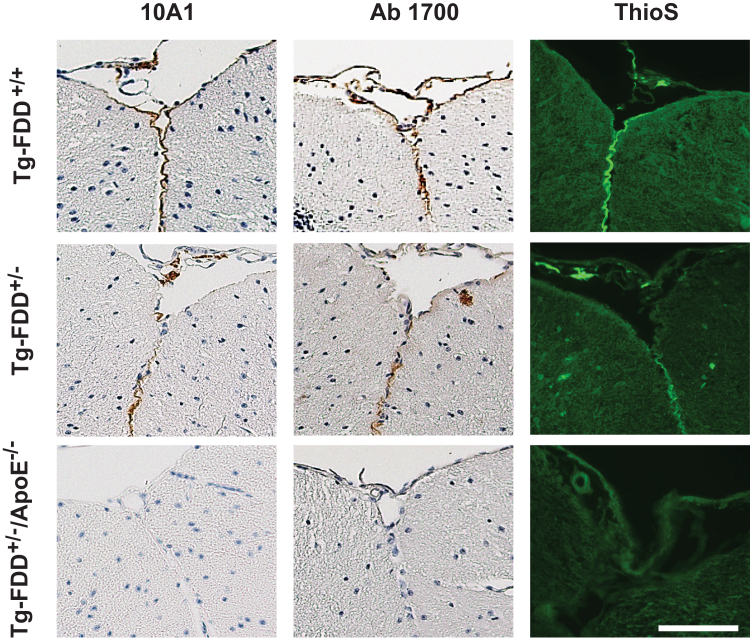
**Amyloid deposition in Tg-FDD mice at 18 months of age.** Typical cerebellar amyloid deposition in leptomeninges in Tg-FDD mice can be seen using the monoclonal antibody 10A1 and polyclonal antibody 1700 in Tg-FDD^+/+^ and Tg-FDD^+/−^ mice. The deposits are typically Th-S-positive, indicating the presence of fibrillar ADan. Amyloid deposits are not observed in Tg-FDD^+/−^ mice on the ApoE^−/−^ background (Tg-FDD^+/−^/ApoE^−/−^) by immunohistochemistry and by Th-S. The scale bar represents 100 μm. FDD, familial Danish dementia; Th-S, thioflavin-S.

**Figure 4:**
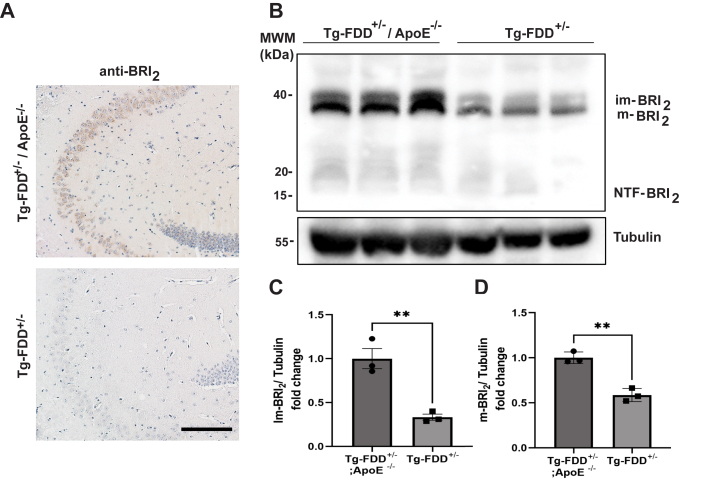
**Accumulation of mutant BRI**_**2**_**in Tg-FDD**^**+/−**^**/ApoE**^**−/−**^**mice.***A*, intracellular accumulation of BRI_2_ in neuronal cell bodies of the CA3 region of the hippocampus of Tg-FDD^+/−^/ApoE^−/−^ mice but not Tg-FDD^+/−^ mice can be observed by immunohistochemistry using an anti-BRI_2_ (anti-ITM2B) ab. Sections were from the hippocampus of 18-month-old mice. The scale bar represents 200 μm. *B*, Western blot analysis of brain homogenates from the hippocampus using the anti-BRI_2_ ab shows a significant accumulation of immature (im-BRI_2_) and mature (m-BRI_2_) forms of BRI_2_ in Tg-FDD^+/−^/ApoE^−/−^ mice. Samples were run in triplicates. Representative blots are shown. *C*, quantification of im-BRI_2_ by densitometry corrected with the densitometry of tubulin (∗∗*p* = 0.0051). *D*, quantification of m-BRI_2_ by densitometry corrected with the densitometry of tubulin (∗∗*p* = 0.0018). All data are expressed as mean ± SD and a *t* test was performed for quantification. FDD, familial Danish dementia.

**Figure S1:**
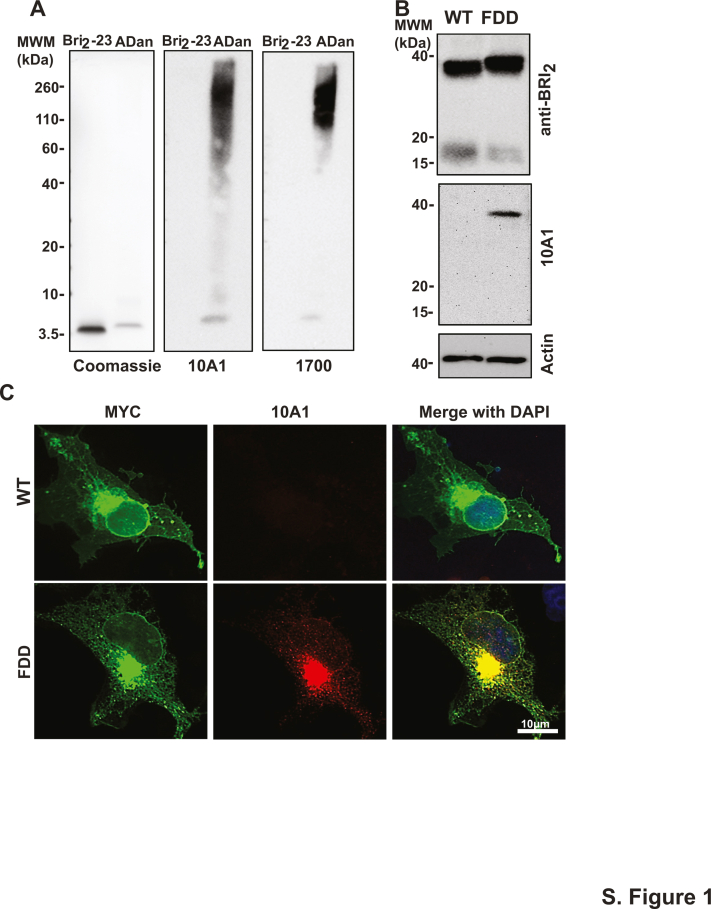


**Figure S2:**
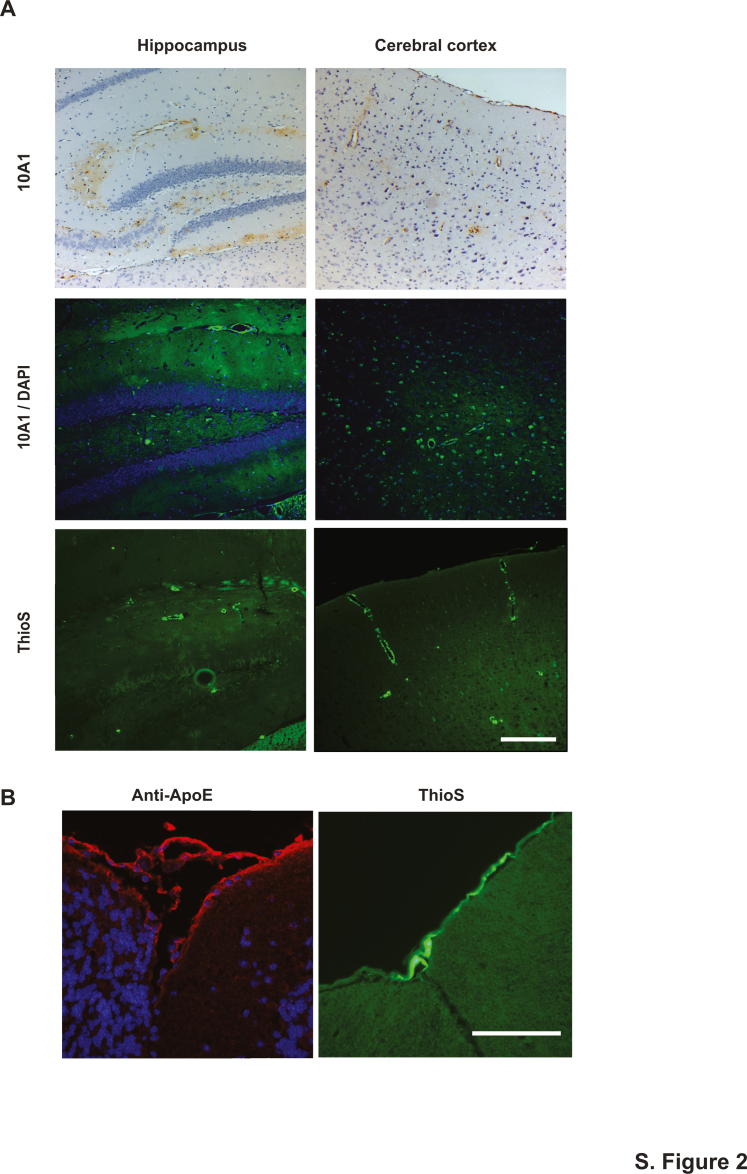


To assess the effect of ApoE on ADan deposition in an *in vivo* model of amyloidosis, we generated mice in which the Danish mutant form of human *BRI*_*2*_ is expressed under the control of the mouse prion protein promoter (Tg-FDD) (10) on an *ApoE* KO (*ApoE*^*−/−*^) background (Tg-FDD^+/−^/*ApoE*^*−/−*^). Mice were analyzed at 3, 6, 9, 12, and 18 months of age. In Tg-FDD mice, ADan amyloid is detectable in animals older than 7 to 8 month of age. At ∼7 months of age, transgenic animals consistently begin to exhibit CAA primarily in pial (leptomeningeal) cerebellar vessels. Immunohistochemical analysis of Tg-FDD^+/−^/*ApoE*^*−/−*^ mice using polyclonal antibodies and monoclonal antibodies (mAbs) specific for the ADan amyloid peptide (Fig. S1) showed that KO of the murine *ApoE* gene led to a complete inhibition of ADan deposition up to the last age analyzed (18 months) (Fig. 3). Thioflavin S (Th-S)–positive ADan deposition in leptomeningeal cerebellar vessels can be seen in age-matched Tg-FDD^+/−^ control mice (Figs. 3 and S2). No parenchymal amyloid deposition was observed in Tg-FDD^+/−^/*ApoE*^*−/−*^ mice. Immunohistochemical analysis using an antibody that recognizes the BRI_2_ precursor protein shows a different pattern of immunoreactivity in the hippocampus of Tg-FDD^+/−^/*ApoE*^*−/−*^ mice, compared to Tg-FDD^+/−^ mice (Fig. 4). The antibody against the N terminus of the BRI_2_ precursor protein labeled neuronal cell bodies in the CA3 region of the hippocampus of Tg-FDD^+/−^/*ApoE*^*−/−*^ mice but not in Tg-FDD^+/−^ mice (Fig. 4*A*). Western blot analyses of protein extracts from the hippocampus showed a statistically significant increase in the levels of the im-BRI_2_ and m-BRI_2_ precursor protein in Tg-FDD^+/−^/*ApoE*^*−/−*^ mice compared to Tg-FDD^+/−^ mice (Fig. 4*B*).

### Gene coexpression analysis defines modules associated with ADan deposition and ApoE

**Figure 5:**
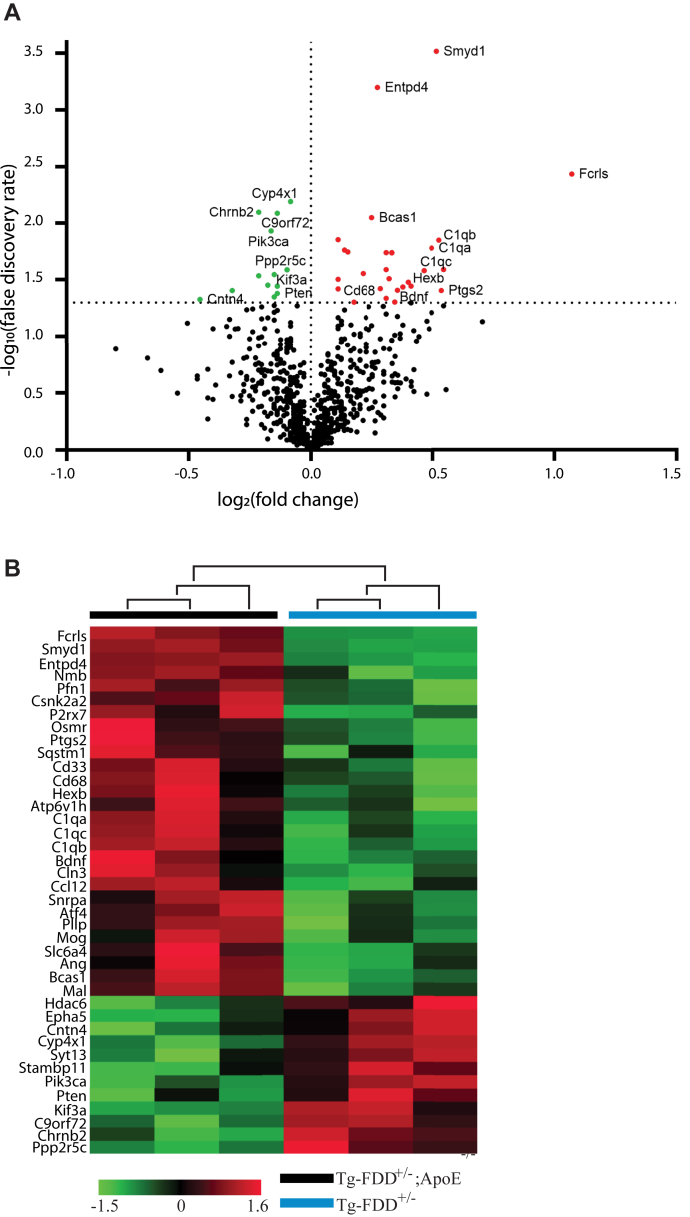
**Transcriptomics analysis of aged Tg-FDD**^**+/−**^**mice *versus* Tg-FDD**^**+/−**^**/ApoE**^**−/−**^**mice.***A*, volcano plot of Nanostring results comparing mRNA isolated from hippocampi of 12-month-old mice, done in triplicate. KO of ApoE expression resulted in the upregulation of 28 genes (*red*) and downregulation of 14 genes (*green*). Genes that are below the cut-off (false discovery rate [FDR] ≤ 1.3.) are marked in *gray*. Some of the highly regulated genes are indicated in the plot. *B*, heatmap of log_2_ fold changes of genes in Tg-FDD^+/−^ mice *versus* Tg-FDD^+/−^/ApoE^−/−^ mice. Vertical lanes represent biological replicates. Genes are color coded as aforementioned. FDD, familial Danish dementia.

**Figure 6:**
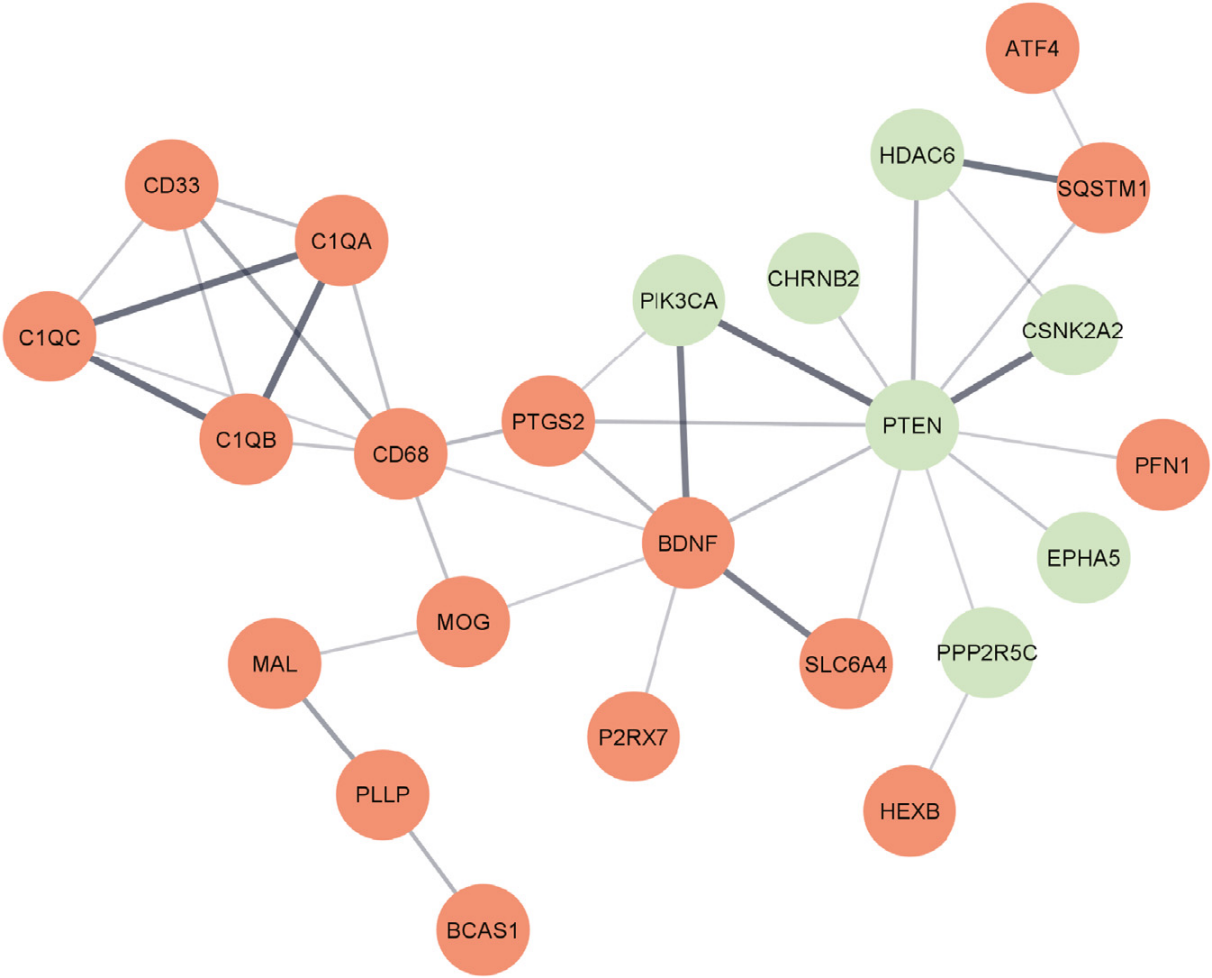
**Prediction of the protein–protein interaction network affected by ApoE KO in Tg-FDD mice.** Network showing the interaction of the top 24 differentially expressed genes, with bold lines indicating the highest confidence score interaction. FDD, familial Danish dementia.

**Figure S3:**
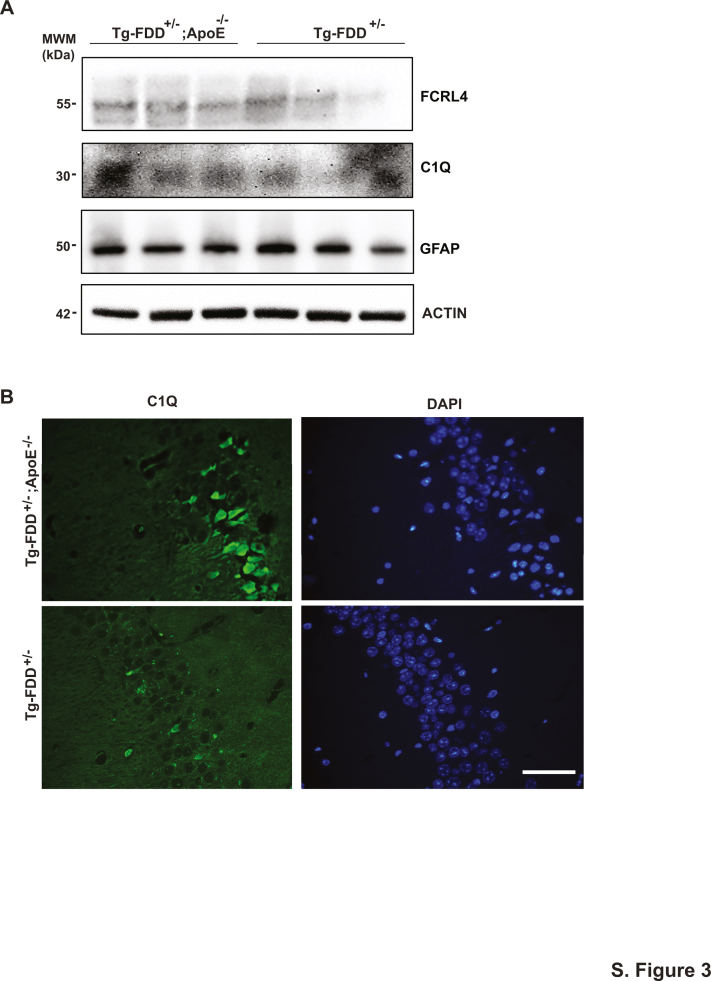


To profile the molecular changes associated with the lack of amyloid deposition in Tg-FDD^+/−^/*ApoE*^*−/−*^ mice, we performed a targeted neuropathological transcriptome analysis using a customized Nanostring nCounter panel containing 770 genes specific for neurodegeneration. Analysis of hippocampal samples from three Tg-FDD^+/−^ and three Tg-FDD^+/−^/*ApoE*^*−/−*^ mice at 12 months of age show 42 significantly misregulated genes, 14 downregulated genes, and 28 upregulated genes (Fig. 5, Table S1 and Fig. S3). Hierarchical clustering analysis showed distinct cluster separation between Tg-FDD^+/−^/*ApoE*^*−/−*^ and Tg-FDD^+/−^ mice (Fig. 5*B*). Network analysis using STRING software to visualize protein–protein interactions of most upregulated and downregulated genes identified four regulated pathways with a high significance score (Fig. 6). The top gene networks identified by genes misregulated in Tg-FDD^+/−^/ApoE^−/−^ mice compared to Tg-FDD^+/−^ mice include autophagy: Hexb (1.3-fold, *p* = 0.03), Cd68 (1.3-fold, *p* = 0.03); activated microglia: Ptgs2 (1.5-fold, *p* = 0.03), C1qb (1.4-fold, *p* = 0.01), C1qc (1.4-fold, *p* = 0.02), C1qa (1.4-fold, *p* = 0.01), Cd68 (1.3-fold, *p* = 0.03); angiogenesis: Ptgs2 (1.5-fold, *p* = 0.03), C1qb (1.4-fold, *p* = 0.01), C1qc (1.4-fold, *p* = 0.02), C1qa (1.4-fold, *p* = 0.01); vesicle trafficking: Ptgs2 (1.5-fold, *p* = 0.03), Cntn4 (-1.4-fold, *p* = 0.04) (Fig. 6).

## Discussion

ApoE has been found present in amyloid deposits in AD and other cerebral and systemic amyloidoses, suggesting a general role for ApoE in amyloid diseases as a pathological chaperone (18, 19). Numerous studies have focused on the direct interaction of Aβ with ApoE and the formation of Aβ and ApoE complexes (33, 34); however, whether similar mechanism(s) may be at play in other amyloids remains to be determined. Like what has been seen in AD, ApoE has been found closely associated with ABri and ADan parenchymal and vascular deposits in FBD and FDD, respectively (35), with Aβ immunoreactivity, mainly in a perivascular position, also present in patients with FDD (3, 7).

In the present study, we determined whether the presence of different ApoE isoforms influence the aggregation of synthetic ADan *in vitro*. ADan-amyloid formation kinetics showed a classical sigmoidal curve, with a lag phase, an elongation phase, and a plateau (saturation) phase. The addition of the different ApoE isoforms to ADan led to a pronounced decrease of Th-T incorporation, with lower plateau levels, but without modifying lag-phases. Thus, in our *in vitro* system, ApoE did not modify exponential fibril growth (elongation phase) but rather modified the level of the plateau, which may be caused by a shift to nonfibrillar structures. Future studies will determine whether the same result is obtained using lipidated ApoE instead of recombinant ApoE. No effect was observed when assessing the Bri_2_-23 peptide in the same assay. The role of ApoE in ADan aggregation *in vivo* was assessed using a cohort of mice expressing the Danish mutant form of human *BRI*_*2*_ on an *ApoE* KO background. Lack of ApoE in Tg-FDD^+/−^/ApoE^−/−^ mice, examined up to 18 months of age, prevented completely the age-dependent deposition of ADan previously observed in Tg-FDD mice (10). KO of *ApoE* in Tg-FDD^+/−^/ApoE^−/−^ mice seems to block the conversion of soluble ADan to fibrillar ADan in both the brain parenchyma and blood vessels. We did not observe a delay in ADan deposition, although we cannot discard amyloid deposition occurring in animals older than 18 months of age. In some transgenic Aβ models, genetic deletion of the murine *ApoE* gene has been shown to delay Aβ accumulation and amyloid deposition (27). During the neuropathologic examination of mice, we unexpectedly found BRI_2_ accumulation in hippocampal neurons of Tg-FDD^+/−^/ApoE^−/−^ mice. We have previously reported accumulation of mutant BRI_2_ in FBD, FDD, and the Tg-FDD model (9) and speculated that the accumulation of the BRI_2_ precursor may be due to the mutant sequences interfering with the correct folding of BRI_2_ and the enzymatic activity of pro-protein convertases. Alternatively, the mutant sequence may disrupt information required for efficient Golgi exit. Further work may clarify whether KO of ApoE may affect protein processing or protein sorting, leading to intracellular accumulation of BRI_2_.

Total RNA-seq studies from brains of AD individuals showed the involvement of the classical complement pathway (CCP) and the phosphorylation of tau to be associated with AD in an *APOE* genotype-specific manner (36, 37). Molecular profiling of the Tg-FDD^+/−^/ApoE^−/−^ model revealed changes in autophagy, activated microglia, angiogenesis, and vesicle trafficking pathways, with C1q, the first subcomponent of the C1 complex of the CCP of complement activation, as one of the most upregulated genes and Ppp2r5c (the protein phosphatase 2 (PP2) regulatory subunit B'gamma), involved in the dephosphorylation of Tau, as one of the most downregulated genes. A total of 14 significantly downregulated genes and 28 significantly upregulated genes were observed. Among these 28 genes, five of them are highly expressed in microglia (Brain RNA-seq database; http://www.brainrnaseq.org/). The top 5 most significantly upregulated genes detected were Smyd1, Entpd4, Fcrls, Bcas1, and C1q (composed of C1qa, C1qb, and C1qc). Smyd1, a histone methyltransferase, is a transcription factor characterized extensively in hematopoietic cells and cardiac/skeletal muscle (38). Recent work suggests that Smyd1 functions also in neuronal cells as regulators of genes disrupted in different neurodegenerative diseases (38). Smyd1 is expressed in microglia cells and astrocytes and was also observed in endothelial cells where it may have a role in inflammation-triggered signaling in endothelial cells (39). Entpd4, the ectonucleoside triphosphate diphosphohydrolase 4 (or NTPDase4), catalyzes the hydrolysis of nucleoside triphosphates and diphosphates in a calcium- or magnesium-dependent manner. The gene is expressed in many different organs, including the brain, and may play a role in the development of gastric cancer (40). Fcrls, which encodes the Fc receptor-like S, is a scavenger receptor expressed by microglia in mice (41). Fcrls are expressed in multiple brain macrophage subsets (42). Fcrls binds ligands that are nonself or self-altered molecules and remove them by phagocytosis. Bcas1, the breast carcinoma-amplified sequence 1, is specifically expressed in immature oligodendrocytes undergoing maturation and myelination. Bcas1 expression defines a population of early myelinating oligodendrocytes in multiple sclerosis lesions (43) and Bcas1-positive immature oligodendrocytes seem to be affected by α-synuclein–induced pathology in multiple system atrophy (44). C1q is the recognition molecule complex of the innate immune system that initiates the CCP. C1q is secreted by macrophages and may play a role in synapse elimination in healthy brain and in neurodegenerative diseases (45, 46). Recently, it has been reported that in ApoE-deficient mice, oxidized lipids activate the CCP and that all three ApoE isoforms and serum-derived ApoE3 bind C1 and C1q (47). Additional important upregulated genes are Hexb, Cd68, Ptgs2, and Cntn4. Hexb encodes the beta subunit of β-hexosaminidase. Mutations in Hexb lead to developmental problems with seizures and childhood death (48, 49) and may be associated with AD and CAA (50, 51). Recent work in mouse models demonstrated that Hexb heterozygosity leads to neuropathologic changes, consistent with previous reports that described a biochemical relationship between Hexb and AD (42, 52). Cd68 is a marker for macrophage lineage cells, primarily localized to microglia within the brain parenchyma and perivascular macrophages in cerebral blood vessels (53). Cd68 labels lysosomal and endosomal transmembrane glycoprotein of microglia, indicating phagocytic activity, with presence of CD68, MSR-A, and HLA-DR being related to dementia and scores of poor cognitive function in AD (54). Ptgs2 encodes the cyclooxygenase-2 (Cox-2), which is involved in the synthesis of prostanoids. Cox-2 seems to be expressed under pathological conditions and to have detrimental effects in AD pathophysiology and neurodegeneration (55). Cox-2 has been recently to be shown to be critical for the propagation of Aβ and reducing the glycosylation of tau in AD (56). Cntn4, *Contactin 4*, is an Ig cell adhesion molecule (IgCAM) gene, which has been associated with several neuropsychiatric disorders including AD (57). Genome-wide association studies showed a link between Contactin family and dementia and dysregulated expression of Contactin in the postmortem brain tissue of AD patients (58).

The top 5 most significantly downregulated genes detected are Cyp4x1, Chrnb2, 3110043021Rik (C9ORF72), Pik3ca and Ppp2r5c. Cyp4x1, the cytochrome P450 4x1, is expressed at very high levels in human and murine brain but the function of this protein is unknown. Cyp4x1-KO mice gained significantly more body weight on normal lab chow diet compared to control flox mice on the same genetic background and had significantly greater intra-abdominal fat deposits (59). Chrnb2 is the nicotinic cholinergic receptor subunit β-2. Genetic analyses have shown an association between *Chrnb2* and late-onset AD (60). Recently, Chrnb2 has been found to be downregulated in chronic traumatic encephalopathy stage III compared to stage II, suggesting cholinergic-related deficits post-traumatic brain injury in humans (61). 3110043021Rik is the murine homolog of human *C9ORF72*. Hexanucleotide repeat expansions in the *C9ORF72* gene are the leading genetic cause of ALS and frontotemporal dementia (62). In humans, *C9ORF72* transcripts are detectable in most tissues, all brain regions, and the spinal cord. C9ORF72 plays an important role in immune regulation. The expression is particularly high in myeloid cells (in particular in CD14^+^ monocytes, eosinophils, and neutrophils) and lower in lymphoid cells and other tissues (63). Pik3ca is the phosphatidylinositol-4,5-bisphosphate 3-kinase catalytic subunit alpha. PIP3 is the major product of PIK3CA. PIP3, in turn, is required for translocation of protein kinase B (AKT1, PKB) to the cell membrane, where it is phosphorylated and activated by upstream kinases. Mutations in Pik3ca lead to a plethora of disorders, among others oncogenic and vascular and overgrowth syndromes with some diseases having a neurologic phenotype ((64), https://www.omim.org/entry/171834). Ppp2r5c is the protein phosphatase 2 (PP2) regulatory subunit B'gamma. The PP2A-PPP2R5C holoenzyme may specifically dephosphorylate and activate TP53 and play a role in DNA damage-induced inhibition of cell proliferation. PP2A-PPP2R5C may also regulate the ERK signaling pathway through ERK dephosphorylation. PP2A is the major tau phosphatase involved in the phosphorylation of Tau in AD (65).

In summary, our findings support the hypothesis that ApoE plays a major role in amyloid deposition in various amyloid diseases. It also demonstrates the beneficial effect of decreasing ApoE levels on ADan accumulation. Whether different isoforms of ApoE can modulate ADan aggregation in FDD, a genetic disorder comparable to familial AD, remains to be determined. The effect of *ApoE*-ϵ4 on onset age of autosomal dominant AD was confirmed in large pedigrees, suggesting that an increased *ApoE-ε4* gene dosage may promote the development of the familial form of the disease (66, 67). Our results from gene array assays demonstrated that a number of genes related to autophagy, angiogenesis, vesicle trafficking, microglia, the CCP, and tau phosphorylation were dysregulated in the brain of Tg-FDD^+/−^/ApoE^−/−^ mice. The genetic screen emphasizes the significant role for ApoE at the interface of inflammation and neurodegeneration *via* glial-mediated mechanisms (68). Future *in vivo* studies may provide additional information regarding the effect of human ApoE isoforms on ADan deposition *in vivo* and the molecular mechanisms and pathways involved.

## Experimental procedures

### Reagents and antibodies

Dulbecco's modified Eagle's medium medium (#12100046), fetal bovine serum (#26140079), penicillin/streptomycin (#15140122), OptiMEM (#31985062), Lipofectamine (#11668030), PBS (#14190), chemiluminescence kit (#32106), and Imperial protein stain (#24615) were purchased from Thermo Fisher. Th-S (T1892) and Th-T (T3516) were purchased from Sigma. Anti-ITM2B (sc-374362) was purchased from Santa Cruz; anti-ApoE (66830) from ProteinTech; anti-C1Q (ab182451) from Abcam; anti-FCRL4 (PA5-87813) from Invitrogen; anti-GFAP (G3893) from Sigma; and anti-Myc (sc-40) and anti-Tubulin (sc-23948) from Santa Cruz. Anti-ADan (ab 1700) polyclonal antibody has been reported previously (10).

### Synthetic peptides

Bri_2_-23 (EASNCFAIRHFENKFAVETLICS) was synthesized by Thermo Fisher Scientific. ADan (EASNCFAIRHFENKFAVETLICFNLFLNSQEKHY) was synthesized by ERI Amyloid Laboratory. The peptides were reconstituted in hexafluoroisopropanol to a final concentration of 1 mg/ml and incubated for 7 days at room temperature. Stock peptide solutions (1 mM) were prepared in 10 mM dimethyl sulfoxide, followed by dilution to 100 μM in PBS buffer.

### Aggregation assay

Peptide solutions were prepared in PBS with 20 μM Th-T to a final concentration of 30 μM. ApoE isoforms (apoE2, apoE3, or apoE4) were used at a 300 nM concentration. The volume of aggregation was 100 μl for all the samples. All samples were transferred to a black 96-well nonbinding surface microplate with clear bottom. The microplate was transferred to a Cytation C10 reader (Agilent). Fluorescence was measured through the top of the plate and recorded every 15 min for 18 h (excitation 440 nm, emission 495 nm) at 37 °C.

### Antibody generation

mAbs were generated by GenScript USA Inc using the antigen sequence CFNLFLNSQEKHY. C57BL/6 mice were immunized using a conventional immunization strategy. Mice with high serum titers (more than 1:60,000), as determined by an ELISA test, were selected for fusion of spleen cells with myeloma cells type SP2/0. Positive cell supernatants were tested by ELISA. The final positive hybridoma cells secreting antibodies against ADan peptide were stored in −80 °C. Supernatants were stable at 2 °C to 8 °C for up to 1 month but for long term storage; aliquots of the supernatants were stored at -20 °C. The 10A1 mAb was chosen for further characterization.

### Animal model

Tg-FDD mice expressing an FDD-associated human mutant BRI_2_ transgene (10) were crossed with ApoE ^tm1Unc^ mice (69) (The Jackson Laboratory) and obtained hemizygous mice Tg-FDD^+/−^/*ApoE*^*+/−*^ that were subsequently intercrossed to generate Tg-FDD^+/−^/*ApoE*^*−/−*^ mice. All mice were on an C57Bl6/J background. Male and female mice were used for the experiments. Mice were housed at the Indiana University School of Medicine (IUSM) animal care facility and were maintained according to USDA standards (12-h light/dark cycle, food and water ad libitum), in accordance with the Guide for the Care and Use of Laboratory Animals (National Institutes of Health, Bethesda, MD). Animals were anesthetized and euthanized according to IUSM Institutional Animal Care and Use Committee–approved procedures.

### Histological and immunohistochemical studies

Mice were anesthetized and transcardially perfused with cold 0.9% saline. After perfusion, the animals were decapitated; the skulls opened and the brains removed and kept at 4 °C in 4% paraformaldehyde in 0.1 M phosphate buffer, pH 7.2, or immediately frozen at −80 °C. Eight micrometer thick sections were cut from the fixed tissue and stained with the H&E and Th-S methods following published protocols (10, 11). Antibodies were visualized by using horse antimouse/rabbit IgG by the peroxidase–antiperoxidase method utilizing 3,3′diaminobenzidine as the chromogen. Images were acquired with a Cytation C10 confocal imaging reader (Agilent).

### Cell transfection

HEK-293T and COS-7 cells were transfected after reaching 75% cell confluence using a 3:1 ratio of DNA:Lipofectamine in Opti-MEM medium with WT human BRI_2_ sequence or FDD-associated human mutant BRI_2_ cloned into the FUGW lentiviral vector (Addgene) with the addition of an N-terminal MYC tag. Cells were transiently transfected for 5 to 6 h with 2 μg of supercoiled plasmid DNA and analyzed after 24 h.

### Immunofluorescence

For immunofluorescence, COS-7 cells were fixed on coverslips with 4% paraformaldehyde in PBS for 10 min, washed with PBS, and permeabilized with 0.2% Triton X-100. Then, cells were incubated with blocking solution (10% bovine serum albumin in PBS) for 1 h, followed by overnight incubation with primary antibody in blocking solution. Finally, cells were incubated with the corresponding secondary antibodies in blocking solution, washed with PBS, and mounted with a mounting medium with 4′,6-diamidino-2-phenylindole. The images were acquired with Cytation C10 confocal imaging reader (Agilent).

### Western blots

Transfected HEK-293T cells and brain samples were homogenized in lysis buffer (Cell Signaling Technology) and proteins were quantified using the bicinchoninic acid method (ThermoFisher). Samples (synthetic peptides or protein samples) were boiled with gel loading buffer for 5 min at 100 °C and separated in 10% Bis–Tris NuPage gels under denaturing conditions. Gels were stained with Coomassie blue or transferred to nitrocellulose membranes. The membranes were incubated with blocking solution (5% nonfat milk in PBS) for 1 h, then incubated for 1 h with primary antibody. Washings were carried out after each incubation with PBS. Finally, they were incubated with secondary antibody for 45 min. Bands were visualized using a chemiluminescence kit (SuperSignal West Pico, ThermoFisher) according to the manufacturer's specifications. Blots were scanned and quantified using Image J software (U.S. National Institutes of Health).

### RNA isolation and Nanostring analysis

Hippocampal tissue was dissected from Tg-FDD^+/−^ and Tg-FDD^+/−^/*ApoE*^*−/−*^ mice. RNA was extracted using the miRNeasy Kit (Qiagen). RNA concentration was determined using a Nanodrop. RNA and Nanostring reactions were prepared according to the manufacturer’s recommendation (Mouse nCounter, Nanostring Technologies). Briefly, hippocampi from four Tg-FDD^+/−^ and four Tg-FDD^+/−^/*ApoE*^*−/−*^ mice at 12 months of age were used for gene expression analyses. Hundred nanograms total RNA per sample were analyzed using the nCounter mouse Neuropathology Panel. Data were analyzed using the nSolver Analysis Software 3.0 (Nanostring Technologies) and ROSALIND software (https://www.rosalind.bio/). An mRNA was considered differentially expressed if the false discovery rate ≤ 1.3 or *p* value < 0.05.

### Statistical analysis

Data are shown as mean ± SD and were considered statistically significant at *p* < 0.05 with GraphPad Prism 7.0 (GraphPad Software Inc).

## Data availability

All the data described in the article are contained within the article.

## Supporting information

This article contains supporting information.

## Conflict of interest

The authors declare that they have no conflicts of interest with the contents of this article.

## Supporting information

FDD-1827HP

FDD-1828HP

FDD-1837HP

FDDApoEKO-71HP

FDDApoEKO-72HP

FDDApoEKO-73HP

Hp Nanostring

NS Mm NeuroPath
