# Supplementary material for: Lack of ApoE inhibits ADan amyloidosis in a mouse model of familial Danish dementia
Source: J Biol Chem. 2022 Nov 25;299(1):102751. doi: 10.1016/j.jbc.2022.102751 (PMC9792896; doi:10.1016/j.jbc.2022.102751)
Supplement: Table S1 [file mmc9.docx]

**STable 1**: List of regulated genes (nCounter Neuropathology Panel) from hippocampus of Tg-FDD^+/-^/ApoE^-/-^ vs. Tg-FDD^+/-^/ ApoE^+/+^ mice.

| **Gene Name** | **Fold change** | **P value** |
| --- | --- | --- |
| Smyd1 | 1.43 | 0.0003026 |
| Entpd4 | 1.21 | 0.00063254 |
| Fcrls | 2.1 | 0.00364949 |
| Apoe | -83.7 | 0.00507571 |
| Cyp4x1 | -1.06 | 0.00641742 |
| Chrnb2 | -1.16 | 0.00804433 |
| 3110043O21Rik | -1.1 | 0.00823115 |
| Bcas1 | 1.19 | 0.00891445 |
| Pik3ca | -1.12 | 0.01174684 |
| Csnk2a2 | 1.08 | 0.01400871 |
| C1qb | 1.44 | 0.01408129 |
| C1qa | 1.41 | 0.01651763 |
| Snrpa | 1.1 | 0.01729284 |
| Pfn1 | 1.11 | 0.01801196 |
| Mal | 1.26 | 0.01809558 |
| P2rx7 | 1.24 | 0.01815103 |
| Atf4 | 1.26 | 0.01819578 |
| Ccl12 | 1.46 | 0.02556913 |
| Cd33 | 1.24 | 0.02559013 |
| Ppp2r5c | -1.07 | 0.02583096 |
| C1qc | 1.38 | 0.02608808 |
| Nmb | 1.16 | 0.02786671 |
| Kif3a | -1.11 | 0.02845941 |
| Epha5 | -1.16 | 0.02920895 |
| Pllp | 1.25 | 0.0308631 |
| Sqstm1 | 1.08 | 0.03135236 |
| Slc6a4 | 1.32 | 0.03310012 |
| Stambpl1 | -1.13 | 0.03525654 |
| Osmr | 1.33 | 0.03586532 |
| Hdac6 | -1.1 | 0.03602546 |
| Hexb | 1.3 | 0.03650922 |
| Ang | 1.22 | 0.03762117 |
| Atp6v1h | 1.08 | 0.03808695 |
| Cd68 | 1.28 | 0.03890591 |
| Ptgs2 | 1.45 | 0.03916261 |
| Gdpd2 | -1.25 | 0.03938048 |
| Pten | -1.1 | 0.04170294 |
| Syt13 | -1.11 | 0.04478184 |
| Mog | 1.24 | 0.04592082 |
| Cntn4 | -1.37 | 0.0470161 |
| Bdnf | 1.27 | 0.04951379 |
| Cln3 | 1.13 | 0.04982044 |
